# Creating HIV risk profiles for men in South Africa: a latent class approach using cross‐sectional survey data

**DOI:** 10.1002/jia2.25518

**Published:** 2020-06-26

**Authors:** Ann Gottert, Julie Pulerwitz, Craig J Heck, Cherie Cawood, Sanyukta Mathur

**Affiliations:** ^1^ Population Council Washington DC USA; ^2^ Population Council New York NY USA; ^3^ Epicentre Health Research Pietermaritzburg South Africa

**Keywords:** segmentation, male, multiple sexual partners, transactional sex, alcohol, gender norms, Latent class analysis

## Abstract

**Introduction:**

Engaging at‐risk men in HIV prevention programs and services is a current priority, yet there are few effective ways to identify which men are at highest risk or how to best reach them. In this study we generated multi‐factor profiles of HIV acquisition/transmission risk for men in Durban, South Africa, to help inform targeted programming and service delivery.

**Methods:**

Data come from surveys with 947 men ages 20 to 40 conducted in two informal settlements from May to September 2017. Using latent class analysis (LCA), which detects a small set of underlying groups based on multiple dimensions, we identified classes based on nine HIV risk factors and socio‐demographic characteristics. We then compared HIV service use between the classes.

**Results:**

We identified four latent classes, with good model fit statistics. The **older high‐risk class** (20% of the sample; mean age 36) were more likely married/cohabiting and employed, with multiple sexual partners, substantial age‐disparity with partners (eight years younger on‐average), transactional relationships (including more resource‐intensive forms like paying for partner’s rent), and hazardous drinking. The **younger high‐risk class** (24%; mean age 27) were likely unmarried and employed, with the highest probability of multiple partners in the last year (including 42% with 5+ partners), transactional relationships (less resource‐intensive, e.g., clothes/transportation), hazardous drinking, and inequitable gender views. The **younger moderate‐risk class** (36%; mean age 23) were most likely unmarried, unemployed technical college/university students/graduates. They had a relatively high probability of multiple partners and transactional relationships (less resource‐intensive), and moderate hazardous drinking. Finally, the **older low‐risk class** (20%; mean age 29) were more likely married/cohabiting, employed, and highly gender‐equitable, with few partners and limited transactional relationships. Circumcision (status) was higher among the younger moderate‐risk class than either high‐risk class (*p* < 0.001). HIV testing and treatment literacy score were suboptimal and did not differ across classes.

**Conclusions:**

Distinct HIV risk profiles among men were identified. Interventions should focus on reaching the highest‐risk profiles who, despite their elevated risk, were less or no more likely than the lower‐risk to use HIV services. By enabling a more synergistic understanding of subgroups, LCA has potential to enable more strategic, data‐driven programming and evaluation.

## 1 INTRODUCTION

Men—previously a “blind spot” in an HIV response focused primarily on women and children [1, 2]—are now its frontier. In sub‐Saharan Africa, men experience intractably high HIV incidence, and contribute to high incidence among adolescent girls and young women (AGYW) [3, 4, 5]. Men in the region are also less likely than women to test for HIV, initiate antiretroviral treatment (ART), and be virally suppressed, and are more likely to die of AIDS‐related illnesses [2]. For these reasons, there has recently been intensified interest in reaching more men with comprehensive social and behavior change programming as well as biomedical prevention and treatment services.

Men are not a monolithic group with respect to HIV risk, even in severe epidemics, and those at highest risk are often “hidden” or “hard to reach” [6]. The goals of understanding and reaching subgroups of men most at risk are currently impeded by few effective tools to figure out how to do so. Increasing age is a clear risk factor for HIV acquisition in South Africa, with prevalence among men rising steeply between ages 20 to 40 [5, 7]. Five‐year age bands (e.g., 20 to 24, 25 to 29) are commonly used to establish and monitor HIV prevention and treatment targets, yet this may be insufficiently nuanced since within any age band, there are likely men at both higher and lower risk. Several key behavioral/attitudinal determinants underlie risk for both acquiring and transmitting HIV in high HIV prevalence settings like South Africa. These include having sex without condoms with multiple sexual partners [6, 7], alcohol abuse [7, 8], and inequitable gender norms and unequal relationship power [9, 10, 11, 12]. Age‐disparate and transactional sexual relationships further contribute to heightened HIV transmission from men to women, and particularly AGYW [13, 14, 15]. Studies examining these factors most commonly use regression‐based approaches and yield information about relationships between individual variables. Yet theory suggests that it is, in fact, combinations of factors that synergistically contribute to heightened risk.

Latent Class Analysis (LCA) provides a rigorous, data‐driven approach to identifying a set of “hidden” subgroups/classes characterized by multiple dimensions in survey data [16, p. 157,^17^]. LCA can be thought of as a “person‐centered” approach to data analysis, in contrast to the dominant “variable‐centered” approach described previously with respect to common regression analyses. The latent classes are identified based on patterns of responses on a selected set of variables, that best represent the data [17]. Originally developed in the 1950’s, LCA has been increasingly used in social and behavioral science research over the last decade [16]. Studies employing LCA in the health field have focused on understanding patterns of substance use/abuse, adolescent risk behaviors, mental health, and intimate partner violence [17, 18, 19]. Several recent studies have applied LCA to understanding risk for HIV or STIs [20, 21, 22, 23, 24, 25, 26], as well as patterns of HIV testing [27, 28]. One recent study among AGYW in South Africa employed LCA to profile their types of male partners, finding five distinct partner types that were associated with incident HIV infection [29]. However, to our knowledge no studies among men in sub‐Saharan Africa have employed LCA to better understand their risk for HIV acquisition/transmission.

In this study we used LCA to identify HIV risk profiles among men in Durban, South Africa. Our goal was to develop an approach for profiling subgroups of men in terms of HIV acquisition/transmission risk that is data‐driven, informative for programming, and potentially applicable in other contexts. We conceptualized HIV risk broadly as risk of either acquiring or transmitting HIV, since our intention was to develop profiles that could inform comprehensive programs to prevent both, and most of the behaviors and attitudes we examined underlie both risk of acquisition and transmission. After developing the profiles, we compared use of HIV services between them.

## 2 METHODS

### 2.1 Study population

From May‐September 2017, we administered surveys to 962 men in two peri‐urban informal settlements in eThekwini district (Durban), Kwa‐Zulu Natal (KZN) province, South Africa. KZN has the highest adult HIV prevalence in the country, at 27% [7]. Informal settlements are characterized by high population density, informal housing structures, and scarcity of social services. South Africa’s National Strategic Plan for HIV, TB and STIs 2017 to 2022 identifies people living in informal settlements as a vulnerable population for HIV and STIs, in need of customized and targeted interventions [30].

Eligible participants were between the ages of 20 to 40. Recent population‐based research in the region has suggested that it is ages 25 to 40 years at which men are most likely to acquire HIV, as well as transmit HIV to AGYW (ages 15 to 24) [31].

### 2.2 Procedures

About two‐thirds of men were recruited at “hot spot” venues, and one‐third at HIV service sites. This dual recruitment strategy was intended to capture a sample of likely high‐risk male partners of AGYW, as well as men already attending HIV prevention and treatment services. Hot spot venues were identified by key informants and included drinking establishments, taxi ranks, and university surrounds (e.g., hang‐out spots near university campuses). HIV service sites were both facility‐based (government/NGO clinics) and community‐based (mobile/home‐based/workplace testing). Additional details about recruitment procedures are included in the Data S1.

The survey was administered by a trained interviewer in isiZulu or English and took an average of 45 minutes. The interviewer read out each question to the respondent and then entered his response using a tablet.

### 2.3 Measures

We developed hypotheses *a priori* about HIV risk profiles, based on formative research with key informants in Durban (focus group discussions/in‐depth interviews with HIV program/service staff and community opinion leaders) and known demographic features of the study area (e.g., concentration of post‐secondary institutions and industry/informal labor‐related employment; low marital/cohabiting rates [32, 33]). We chose not to construct separate LCA models for risk of HIV acquisition versus transmission for several reasons. First, as described above, most of the behavioral/attitudinal determinants we examined confer risk for both acquisition and onward transmission, and we wanted to identify profiles that could inform comprehensive programs to prevent both. In addition, restricting samples by self‐reported HIV status was not advisable given likely underreporting of HIV‐positive status and low sample size of HIV‐positive men (n = 84) (for further detail see Data S1).

We identified ten demographic, attitudinal, and behavioral variables we believed would define and distinguish between the hypothesized profiles, informed by the literature described previously regarding key risk factors for HIV acquisition/transmission. It was not informative to include self‐reported HIV‐positive status in the model, since prevalence increased with age per known prevalence patterns among men in KZN [5, 7]. Table 1 includes detailed descriptions of the measures.

**Table 1:** Measures for variables included in LCA models and postestimation analyses

Variable	Measure description
**Socio‐demographics**	
Age	Continuous; based on the question *“What is your age?”*
Marital/cohabiting status	Binary; defined as married or cohabiting versus not, based on the question *“What is your current relationship status?”*
Highest level of education completed	Ordinal; consolidated from six response options into three categories: some secondary or less, secondary, or technical college/university. Based on the question *“What is the highest level of education you have completed?”*
Occupation	Categorical; seven categories, including unemployed. Based on the questions *“Are you currently working”* (with “No” corresponding to the unemployed category) and *“What do you do for work?”*, with 24 response options. The final occupation variable was consolidated into seven categories (including unemployed as one), based on (a) sufficient sample size in each occupational category, and (b) grouping similar occupation types together. Response options with <10 responses, that could not be meaningfully combined with others to form a category making up >5% of the sample, were categorized as ‘other’
**HIV risk factors**	
Endorsement of inequitable gender norms	Binary; based on mid‐point cutoff of a continuous scale score. The continuous variable was measured using an adapted version of the Gender‐Equitable Men’s (GEM) Scale [34], previously validated in South Africa [35]. The final 19‐item scale demonstrated good reliability (ordinal theta = 0.93 [36]). Example items are: *“A man should have the final word about decisions in his home”; “Sometimes a woman needs to be put in her place”;* and *“It is a woman’s responsibility to avoid getting pregnant.”* Response options were agree/partly agree/do not agree. We generated a mean GEM Scale score for each respondent. Then, for clear interpretation (i.e., identifying highly inequitable gender norms), and to reduce the number of continuous variables in the model to improve convergence, we dichotomized scores at the midpoint of the possible range to represent endorsement of more inequitable versus equitable views
Number of sexual partners in the last year	Ordinal; with 3 categories: 0 to 1, 2 to 4, or 5+ sexual partners in the last year. Based on the question *“Over the last 12 months, how many different female sexual partners have you had? If you are not sure of the exact number please give a best guess.”* (Of note, <1% of respondents reported ever having had sex with a man.) Categorizing the number of sexual partners into 0 to 1, 2 to 4, or 5 + helped differentiate two aspects of interest: (a) the class’ prevalence of no partners/monogamous relationships (i.e., 0 to 1 vs. more), and (b) the proportion with a very high number of sexual partners (i.e., 5+ vs. fewer)
Age disparity of relationships	Continuous; calculated as the mean age difference with up to the respondent’s last three non‐marital/non‐cohabiting partners reported on a partner grid (for each, subtracting the partner’s reported current age from the respondent’s age). Most partners were younger; 13.5% of non‐marital/non‐cohabiting partners were older (median of 2 years older; data not shown). Age difference with any marital/ cohabiting partners was not included in the calculation because in KZN marital/cohabiting partners tend to be closer to men’s own age [31], whereas this indicator mainly seeks to capture contribution of age disparity to risk of HIV transmission from men to younger women
Consistent condom use	Binary; with each of up to the last three non‐marital/non‐cohabiting partners reported in a partner grid, defined as reporting ‘always’ (vs. ‘sometimes’ or ‘never’) in response to the question *“For the last 3 months you were having sexual intercourse with [this partner], how often was a condom used?”*
Engaging in transactional relationships in the last year	Categorical; three categories including: none, less resource‐intensive, and more resource‐intensive. This categorization was based on reporting giving at least one item or service (the response categories) ‘mainly so you could start or stay in a sexual relationship’ with a partner [13]. Men who qualified but were married/cohabiting with no other reported partners in the last year, were coded as not having transactional relationships, since transactional sex is commonly defined as involving exchange of sex for material support with non‐marital/non‐cohabiting partners. “Less resource‐intensive” transactional relationships included providing cash/money; drugs, food, cosmetics, clothes, a cell phone, airtime; transportation; or somewhere to sleep for the night. “More resource‐intensive” included providing somewhere to live; support or money for their children or family; or money to pay for debt/loans/school or university fees. Since most men who reported “More resource‐intensive” forms also reported less resource‐intensive forms, to create mutually exclusive categories, “More resource‐intensive” included either only providing more‐resource intensive, or both more‐ and less‐resource intensive
Hazardous drinking	Binary; measured using the concise version of the Alcohol Use Disorders Identification Test (AUDIT‐C) [37], which asks whether, how much, and how often the participant drinks alcohol, with a total score ranging from 0 to 12. We created a binary variable with a score of 4 or above (standard cutoff) indicating hazardous drinking [37]
**HIV service use measures** (for postestimation analyses)	
HIV testing in the last 12 months	Binary; based on response of ≥1 to the question *“In the past 12 months, how many times have you been tested for HIV? Please only include tests for which you received the results. If you don’t know the exact number give a best guess.”* Men who self‐reported being HIV‐positive and initiating antiretroviral therapy over 12 months ago (suggesting they did not need to test for HIV in the last 12 months), were coded as missing
Circumcision status	Binary; based on answering ‘Yes’ to the question *“Have you been circumcised?”* (with validity of the response further confirmed/corrected through a series of follow‐up questions, e.g., age circumcised, type of circumcision, whether considering getting circumcised in the future)
HIV treatment literacy	Discrete variable with values ranging from 0 to 5, based on the number of correct yes/no responses to five questions about antiretroviral treatment: (1) *“Can antiretroviral therapy (ART) help a person with HIV to stay healthy and live longer?”* (Yes = correct) (2*) “Do you think HIV/AIDS can be cured?”* (Yes = incorrect) (3) *“Are there any special drugs that a doctor can give a pregnant woman infected with HIV/AIDS to reduce the risk of transmission to the baby?”* (Yes = correct) (4) *“Can taking breaks from antiretroviral therapy (ART) make it work better in the long term?”* (Yes = incorrect) (5) *“Can taking antiretroviral therapy (ART) reduce the risk of transmitting the HIV/AIDS virus to another person?”* (Yes = correct)
Current antiretroviral therapy (ART) use (among HIV‐positive respondents)	Binary; based on responding ‘Yes’ to the question *“Are you currently taking antiretroviral therapy (ART)?”*

### 2.4 Analysis

#### 2.4.1 Model definition

All analyses were conducted using Stata v15 [38]. The LCA followed an iterative process that involved constructing a series of models and refining the variables included. We began by fitting 1‐ to 5‐class models. With each model, we assessed identification, interpretability, overall model fit, and each indicator’s ability to differentiate among the classes (e.g., >5% difference between most classes). If an indicator consistently produced similar probabilities across all of the classes, it was excluded from the model.

Final model selection was based on identification and relative‐fit statistics (for details see Table 4 and the Data S1), as well as interpretability. For interpretability, we considered whether the latent classes made logical sense and were distinct from each other [17]. The final model yielded probabilities for class membership, and, for each class, item response probabilities for each indicator.

#### 2.4.2 Postestimation analyses

For postestimation analyses, each respondent was assigned to a class based on their highest posterior latent class probability. We then assessed associations between class membership and four variables (for which measures are described in Table 1): HIV testing in the last year, ever‐circumcised, treatment literacy score, and current ART use (among HIV‐positive men). Poisson regression was used for treatment literacy (a count variable). For the rest, generalized linear models with a binomial distribution and log link function were used to compute prevalence ratios. This is a recommended approach for binary outcomes characterized by relatively high prevalence [39, 40] (a sensitivity analysis using logistic regression yielded nearly identical results). We adjusted models for type of hot spot venue/service site. We did not adjust for demographic characteristics because those characteristics were included in the LCA model.

### 2.5 Ethics

This study was approved by the Institutional Review Boards at the Population Council and University of Kwa‐Zulu Natal. We obtained written informed consent from all participants.

## 3 RESULTS

A total of 962 men participated in the survey. The response rate was 97.3% (18 refusals) at hot spot venues and 99.1% at HIV service sites (3 refusals); this reflects the proportion agreeing to participate after entering the study tent and being read a description of the study. Fifteen respondents were dropped from the analysis since they were missing values for three or more of the ten variables included in the initial LCA models. This resulted in a final sample size of 947, 638 recruited at hot spot venues and 309 at HIV service sites.

Sample characteristics are presented in Table 2. The mean age was 28 years (range 20 to 40). Fifteen percent of participants were married or cohabiting, similar to documented marital/cohabiting rates in urban informal settlements in the country [33]. Most had completed secondary school (56%), and over one‐third (39%) were unemployed. Among employed men, the most common occupation was taxi/bus driver (25%), likely due to having recruited partly at taxi ranks. Other common occupations included being a factory/construction worker, informal laborer, service industry worker, and small business owner/entrepreneur.

**Table 2:** Sample characteristics (n = 947)

	n/Mean	%/SD
**Socio‐demographic**		
Age	27.7 years	5.5 years
Married/cohabiting	146	15.4%
Education (highest completed)		
Some secondary or less	215	22.7%
Secondary	529	55.9%
Technical college/University	203	21.4%
Occupation		
Unemployed	370	39.1%
Taxi/bus driver	235	24.8%
Factory/construction worker	72	7.6%
Informal labor	51	5.4%
Service industry	63	6.7%
Small business/entrepreneur	48	5.1%
Other occupation	108	11.4%
**Normative gender attitudes**		
Inequitable views towards gender norms	235	24.8%
**HIV risk behaviors**		
Number of sexual partners in last year		
0 to 1	277	29.3%
2 to 4	452	47.7%
5+	218	23.0%
Age difference with last 3 partners (mean years younger)	3.5 years	3.7 years
Transactional relationships		
None	416	43.9%
Less resource‐intensive	405	42.8%
More resource‐intensive	115	12.1%
Hazardous drinking	486	51.3%

SD, Standard deviation. For each variable, missingness was < 2%. Overall missingness was < 1%. Per Stata v15 standard procedures, missing values were imputed based on equation‐wise deletion, which uses valid responses from other variables to estimate missing values [41].

### 3.1 Latent class solution

Initial variable re‐coding (Table 1) was performed for about half of the variables to eliminate collinearity, ensure adequate cell size per response category, and/or condense certain continuous variables to enable model fit. We fit a one‐class to a five‐class LCA model; each of these solutions was identified except for the five‐class. The only variable dropped due to low variability across the latent classes was consistent condom use (with up to last three non‐marital/non‐cohabiting partners), which was consistently at about 20% for each class (data not shown).

We found four latent HIV risk classes (Table 3). The four‐class model was selected because the classes were distinct from each other in terms of item response probabilities and were more interpretable than other class solutions. The model also had good fit statistics (AIC = 21,275; BIC = 22,079; entropy = 0.76) (Table 4). For ease of reference, we labeled the four classes Older high‐risk, Younger high‐risk, Younger moderate‐risk, and Older low‐risk. Younger/older age was chosen to include in the label due to the discrepancy in ages between classes (for simplicity classified as below/above the sample mean of 28 years, within the sample’s limit of 20 to 40 years). Risk level was chosen to denote which classes may be more important to reach with HIV prevention/care services, and since for each class most or all risk characteristics represented a consistent level of risk for HIV acquisition/transmission.

**Table 3:** HIV risk profiles among men (n = 947)

	Class membership (probability)			
	Older high‐risk (19.6%)	Younger high‐risk (24.1%)	Younger moderate‐risk (36.4%)	Older low‐risk (19.9%)
	Item response probabilities			
**Socio‐demographic**				
Age	35.9 years	27.2 years	22.5 years	29.4 years
Married/cohabiting	37.1%	7.8%	3.6%	24.8%
Education (highest completed)				
Some secondary or less	35.7%	20.0%	16.9%	23.8%
Secondary	46.5%	66.2%	51.0%	61.5%
Technical college/University	17.8%	13.8%	32.1%	14.7%
Occupation				
Unemployed	16.8%	20.9%	73.5%	20.2%
Taxi/bus driver	30.0%	35.8%	11.5%	30.7%
Factory/construction worker	11.3%	12.4%	2.5%	7.6%
Informal labor	7.3%	5.1%	1.2%	11.4%
Service industry	10.2%	5.3%	3.3%	10.8%
Small business/entrepreneur	8.7%	9.4%	2.0%	2.0%
Other occupation	15.7%	11.1%	6.0%	17.3%
**Normative gender attitudes**				
Inequitable views towards gender norms	26.5%	38.8%	25.5%	6.4%
**HIV risk behaviors**				
Number of sexual partners in last year				
0 to 1	35.7%	4.2%	27.7%	56.1%
2 to 4	46.7%	54.3%	49.8%	37.1%
5+	17.6%	41.5%	22.5%	6.8%
Age difference with last 3 partners (mean years younger)	8.0 years	3.6 years	1.1 years	3.6 years
Transactional relationships				
None	49.7%	6.7%	49.3%	76.7%
Less resource‐intensive	29.8%	75.7%	44.7%	14.2%
More resource‐intensive	20.5%	17.6%	6.0%	9.1%
Hazardous drinking	58.7%	72.6%	41.2%	39.3%

**Table 4:** Model fit statistics and class assignment diagnostics

Goodness‐of‐fit statistics						
Model	Observations	Log likelihood	DF	AIC	BIC	Entropy
1‐Class	947	−11363.3	19	22764.6	22856.8	—
2‐Class	947	−1002.1	37	22078.2	22257.7	0.79
3‐Class	947	−10878.1	55	21866.1	22133.0	0.78
4‐Class	947	−10789.2	73	21724.5	22078.8	0.76

Assignment accuracy diagnostics				
Classes	Probability of class membership	Proportion assigned to class	AvePP	OCC
Older high‐risk	0.196	0.196	0.92	47.17
Younger high‐risk	0.241	0.250	0.81	13.43
Younger moderate‐risk	0.364	0.360	0.91	17.67
Older low‐risk	0.199	0.193	0.82	18.34

AIC, Akaike Information Criteria; AvePP, Average (mean) Posterior Probability of Assignment, ≥0.70 indicates high assignment accuracy [42]; BIC, Bayesian Information Criteria, with lower values signifying a better fit [17]; DF, degrees of freedom; OCC, Odds of Correct Classification, OCC > 5 represents high assignment accuracy [42].

The closer the entropy value is to 1, the stronger the separation between classes [43].

The 4‐class model does not meet the conditional independence assumption; however, experts have emphasized that this assumption is more difficult to meet when classifying based on behavioral indicators, and that conditional independence must be balanced with interpretability [44, 45].

We did not calculate the Likelihood‐Ratio test for each model, since this test is based on the chi‐squared statistic which requires observed and expected values and can only be used when all indicators are categorical [41].

The **older high‐risk** class comprised one‐fifth (19.6%) of the sample and had the highest mean age of the sample (35.9 years). This class was the most likely of the classes to be married/cohabiting (37.1%) and least likely to be unemployed (16.8%). The occupation with the highest probability was taxi/bus driver (30.0%). Men in this class had a 26.5% probability of endorsing inequitable gender norms, and 46.7% and 17.6% probabilities, respectively, of having 2 to 4 partners and 5 + sexual partners in the last year. Among the classes, these men’s relationships had the greatest age‐disparity (on average, 8.0 years younger) and were more resource‐intensive transactional in nature (20.5%). Finally, this class had a high probability (58.7%) of reporting hazardous drinking.

The **younger high‐risk** class (24.1 % of the sample) were relatively young (mean age 27.2 years) and had a low likelihood of being married/cohabiting (7.8%). They had a 66.2% probability of having completed secondary school and a 20.9% probability of being unemployed and were most likely to be taxi/bus drivers (35.8%) and more likely than other classes to be factory/construction workers (12.4%). Of the four classes, this class was most likely to endorse inequitable gender norms, at 38.8%. They also had the highest probabilities of most risk behaviors, at 54.3% for having 2 to 4 partners in the last year and 41.5% for having 5+; on average their partners were four years younger than themselves. They had the highest likelihood of engaging in transactional relationships, primarily those less resource‐intensive in nature (75.9%), as well as hazardous drinking (72.6%).

The **younger moderate‐risk** class was the most prevalent class of the sample (36.4%), with an average age of 22.5 years. Among the classes, they were the least likely to be married/cohabiting (3.6%) and the most likely to have completed technical college/university (32.1%). However, they also had the highest probability (73.5%) of being unemployed (although about half of those unemployed were still in school (data not shown)). The likelihood of endorsing inequitable gender norms was similar to the older high‐risk class, at 25.5%. This class had a high probability of having multiple sex partners (49.8% with 2 to 4; 22.5% with 5+), but the mean age difference with these partners was only one year. They also had a relatively high probability (50.7%) of having transactional relationships, mostly less resource‐intensive in nature (44.7%), but a lower probability than the high‐risk classes of engaging in hazardous drinking (at 41.2%).

Finally, the **older low‐risk** class (mean age of 29.4) was about as prevalent in our sample as the older high‐risk class, both at about 20%. They had a 24.8% likelihood of being married/cohabiting, and a 20.2% likelihood of being unemployed. Of the four classes, men in this class were least likely to endorse inequitable gender norms (6.4%), have multiple sexual partners (56.1% had 0 to 1 partner), engage in transactional relationships, and report hazardous drinking.

Of note, there were no significant differences between the classes in terms of recruitment strategy (i.e., hot spot venue vs. HIV service site) nor location (i.e., the two informal settlements). We conducted a sensitivity analysis in which we restricted the sample to respondents who did not report being HIV‐positive. The resulting LCA model was markedly similar to the full‐sample model (see Data S1 for details), therefore we chose the latter as the final model.

**Figure 1:**
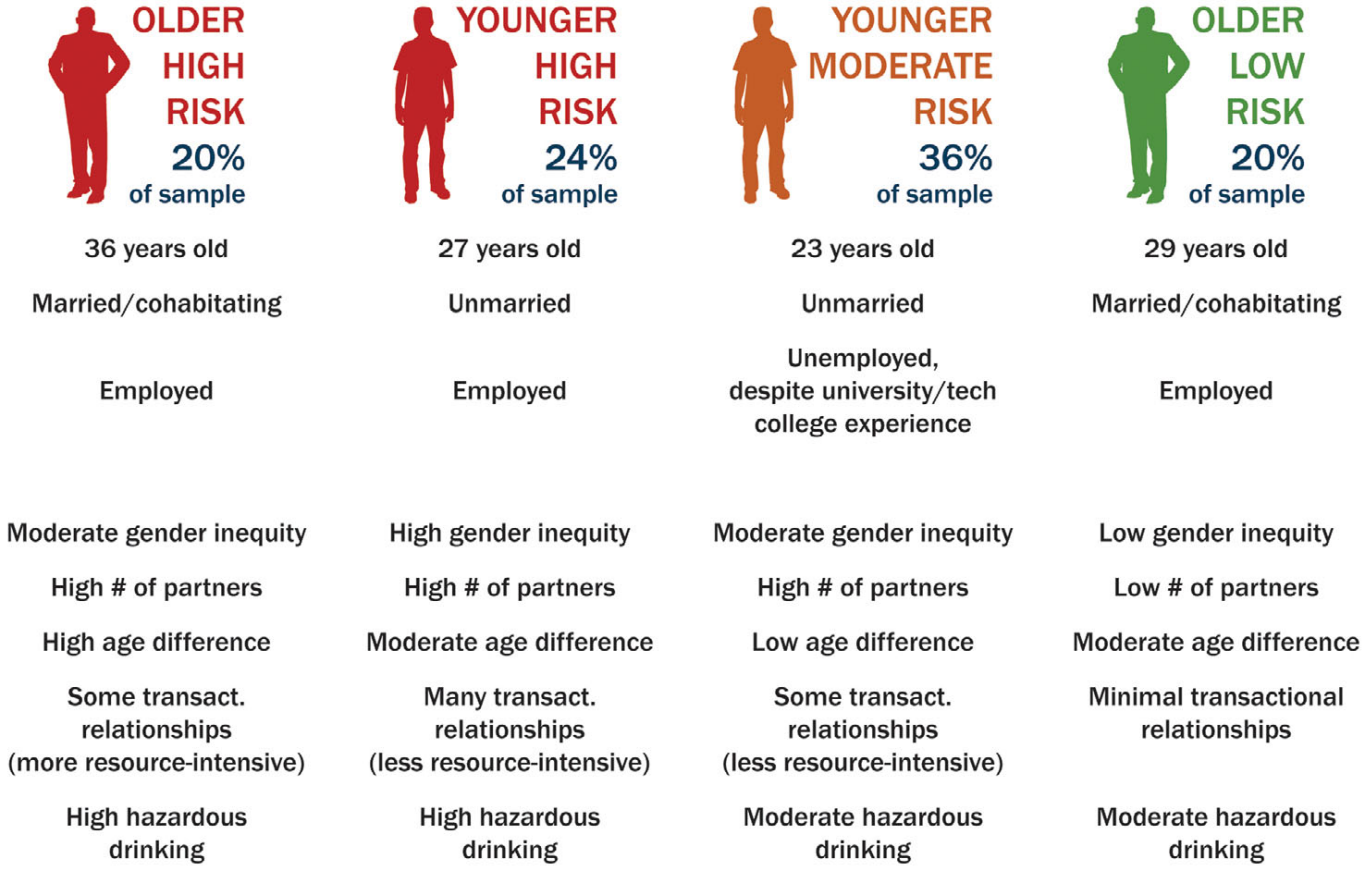
Graphic summarizing men’s HIV risk profiles, Durban, South Africa.

Figure 1 is a visual that aims to provide an easily‐interpretable snapshot of the four classes, to facilitate translation into programmatic implications.

### 3.2 Associations between latent class membership and use of HIV services

For postestimation analyses, each respondent was assigned to a class. Class assignment diagnostics (Table 4) suggested a low chance of misclassification; for example, the average posterior probabilities of class assignment ranged from 0.81 to 0.92.

Three‐quarters (74.3%) of men assigned to the younger moderate‐risk class reported being circumcised (Table 5), compared with 55.6% among the younger high‐risk (aPR 0.75, 95% CI 0.68, 0.84, *p* < 0.001) and 45.1% among the older high‐risk (aPR 0.61, 95% CI 0.51, 0.73, *p* < 0.001). There was no association between class membership and levels of HIV testing in the last year (at 64 to 68% across classes) or HIV treatment literacy (at 3.4 to 3.7 on a scale of 0 to 5). Among HIV‐positive men, current ART use was similar between classes at 89 to 96%.

**Table 5:** Associations between latent class membership and HIV service use

	Tested for HIV in last 12 months (n = 513)^b^		Circumcised (n = 939)		HIV treatment literacy score (range 0 to 5) (n = 944)		Currently taking antiretroviral therapy (n = 80)^c^	
	n (%)	aPR (95% CI)	n (%)	aPR (95% CI)	Mean ± SD	aIRR (95% CI)	n (%)	aPR (95% CI)
*Full sample*	337 (65.7%)	–	576 (61.3%)	–	3.55 ± 1.00	–	73 (91.3%)	–
Older high‐risk	57 (65.5%)	0.97 (0.79, 1.19)	83 (45.1%)	0.61 (0.51, 0.73)***	3.56 ± 1.00	1.00 (0.96, 1.04)	27 (90.0%)	–^d^
Younger high‐risk	89 (64.0%)	0.95 (0.82, 1.10)	130 (55.6%)	0.75 (0.68, 0.84)***	3.38 ± 1.06	0.94 (0.88, 1.01)	16 (88.9%)	–^d^
Younger moderate‐risk	138 (67.7%)	ref	252 (74.3%)	ref	3.57 ± 1.03	ref	9 (90.0%)	–^d^
Older low‐risk	53 (63.9%)	0.94 (0.78, 1.13)	111 (61.0%)	0.83 (0.71, 0.95)*	3.72 ± 0.81	1.05 (0.98, 1.11)	21 (95.5%)	–^d^
*Overall p‐value* ^a^	0.89		<0.001		0.18		0.88	

Analyses adjusted for recruitment site.

^a^ Overall *p*‐value represents overall statistical significance of difference between groups, based on Pearson’s chi‐square test.

^b^ Among venue‐based sample only, since service‐based sample included many coming for HIV testing. Excluded men who reported initiating ART (i.e., were diagnosed) over 12 months ago.

^c^ Four of the 84 HIV‐positive men did not provide a valid response regarding current ART use.

^d^ Small sample sizes for each class precluded testing significance of differences between them.

*p < 0.05, **p < 0.01, ***p < 0.001; significance of comparisons with the reference category (ref; selected based on youngest mean age of the latent class).

aIRR, adjusted incidence rate ratio; aPR, adjusted prevalence ratio; CI, confidence interval; SD, standard deviation.

## 4 DISCUSSION

This study demonstrated the value of applying LCA to advance HIV prevention research in sub‐Saharan Africa, by enabling a more synergistic understanding of subgroups of men. We identified four distinct HIV risk profiles among men in Durban, with different socio‐demographic characteristics and risk factors. Two of the four profiles (one younger, one older) had markedly higher likelihoods of HIV risk factors than the lower‐risk groups and, despite this, were less or no more likely to use services.

LCA results clearly distinguished between the classes. There were large differences in most indicators and substantial consistency of risk factor probabilities within each class. Including demographic, attitudinal, and behavioral indicators within the LCA model (as opposed to just risk behaviors) helped develop more interpretable and nuanced profiles, which could prove more useful to intervention planning. Taken together, these findings add to a developing evidence base demonstrating the benefits of a multidimensional approach to modeling HIV acquisition/transmission risk [46], and could help identify subgroups of men we need to prioritize for prevention programs and services. Complementing these findings with qualitative research and consultations with local stakeholders could help funding agencies and implementing partners further translate the profiles into concrete decisions about where and how to reach these groups and with what types of programming/services.

The popular discourse and peer‐reviewed literature have focused much attention on older men with financial means having much younger female partners, with relationships motivated by transaction and power imbalances—often called “sugar daddies” or “blessers” [47, 48, 49, 50]. Recent evidence from phylogenetic analyses in South Africa suggests it is men of approximate ages 25 to 40, on average 8.7 years older than their non‐marital/non‐cohabiting partners, who contribute the most incident infections among AGYW [31]. One‐fifth of our sample of men ages 20 to 40 generally fit the “sugar daddy” description; thus, this group still requires tailored prevention activities. However, it was in fact younger men—one‐quarter of the sample—who were found to reflect the highest levels of risk.

The younger high‐risk class should be a particular focus of prevention efforts, as they had the highest probabilities across nearly all risk indicators, as well as suboptimal HIV service uptake. With a mean age of 27, around the peak age for incidence among men and male partners of AGYW [5], strategic HIV testing, with immediate linkage to care, as well as voluntary medical male circumcision (VMMC), are all priorities for this group. Workplaces—such as taxi ranks, factories, and construction sites—could be potential community testing and VMMC promotion sites for younger high‐risk men.

The most prevalent profile among our sample was younger moderate‐risk men, at 36%, who were commonly university/technical college students/graduates and unemployed. Their predominant risk factor for acquiring/transmitting HIV was their high probability of having multiple sexual partners (including 5 + partners); this was tempered by a lower mean age difference with partners and lower probability of transactional relationships than the younger high‐risk class. Finally, the older low‐risk profile had markedly low probabilities of risk behaviors compared with the other profiles. Further research with this group could provide insights into protective factors that could aid in designing interventions for the others.

Endorsement of inequitable gender norms was markedly more likely among the younger high‐risk group and less likely among the older low‐risk group. This is in line with previous research demonstrating causal links between inequitable gender norms/unequal relationship power and HIV risk behaviors/incidence, and suggests that changing gender norms should be prioritized within prevention programming [9, 10, 12].

It is important to recognize that the sampling strategy was formulated to intentionally locate men at high risk of HIV by focusing on informant‐identified hot spot venues. This strategy was effective, as the sample as a whole reported very high levels of risk. Thus, concentrating future prevention activities at such venues could help reach more of the “right” men with HIV prevention interventions. Yet, equally important is that even within this sample, it was possible to identify both higher‐risk and lower‐risk groups. This suggests that using approaches like LCA can help target limited resources in contexts where much of the population is at risk, as well as identify high‐risk subpopulations within large samples.

Another unique approach of our study is comparing HIV service uptake across profiles. With this information, program planners and implementers can create a more nuanced picture – beyond five‐year age bands, for example – of who is being reached with each type of service. To maximize prevention of HIV acquisition and/or transmission, it is particularly important to reach those at highest risk with services. Yet among men in our study, the highest risk profiles did not use services any more than the lower risk profiles. And, with the exception of current ART use, HIV service use and consistent condom use were suboptimal for all groups, per the current National Strategic Plan for HIV, TB and STIs [30].

LCA could be a useful tool for future HIV prevention efforts among men in several ways. Using LCA to profile men in terms of their HIV acquisition/transmission risk is an approach that could be replicated in other geographic locations. Applying LCA to existing survey data is also possible. Such surveys, many of which include similar variables to those in the present study, have been conducted in numerous high‐prevalence locations that would benefit from a better understanding of at‐risk populations. This approach also has potential in terms of monitoring and evaluation. For example, using multiple cross‐sectional surveys, one could monitor changes in HIV service use over time by profile or see how an intervention differentially impacted each profile.

This study had several limitations. First, survey responses were based on self‐report, potentially introducing social desirability bias. Second, the cross‐sectional nature of the data precludes inferences that latent class membership was causally associated with HIV service use. Third, the extent to which the risk profiles are linked to actual HIV acquisition or transmission remains unclear. Fourth, the conditional independence assumption was not met for the final model, although we do not believe this invalidates the model (see note below Table 4). Fifth, by using posterior probability to assign class membership, it is possible that some misclassification occurred. Additionally, for the postestimation analysis for last‐year HIV testing, we coded as missing HIV‐positive men who initiated ART > 1 year ago as a proxy for diagnosis >1 year ago (not captured on the survey); however this may have missed some ineligible men. Finally, the study was limited to two informal settlements in Durban and to men recruited at hot spot venues and HIV service sites. Therefore findings, including the prevalence of each latent class, may not be generalizable to all men ages 20 to 40 in Durban or other locations in the region. In addition, studies taking a different approach to identifying profiles may find different profiles than those identified in the present study.

## 5 CONCLUSIONS

Study findings elucidate a nuanced picture of who the right men are to reach with HIV prevention and treatment programs in Durban, South Africa, and how those programs could be tailored for subgroups representing varying levels of HIV risk. In particular, it is critical to reach both the younger and older high‐risk groups with HIV prevention programming and services, grounded in an understanding of the different characteristics of each (e.g., younger high‐risk as employed, non‐university‐affiliated, unmarried, with ubiquitous hazardous drinking and minimally‐resource‐intensive transactional relationships).

The extent to which the HIV risk profiles we found differ for men in other geographic locations in South Africa and/or other countries remains to be seen, presenting a rich area for future research. It may be that similar patterns of risk, and/or notable differences, will emerge across contexts. Future studies should also explore comparative advantages of having separate models for risk of HIV acquisition versus transmission, particularly if biological endpoints are available, and employ longitudinal designs to track change over time. In sum, LCA is a promising data‐driven tool for profiling population sub‐groups, that could enable more strategic design and evaluation of HIV prevention, care and treatment programs.

## COMPETING INTERESTS

The authors declare no conflicts of interest.

## AUTHORS’ CONTRIBUTIONS

Study conception: AG JP SM; study design: AG JP SM; protocol development: AG JP SM CC; data collection: CC AG; data analysis and manuscript preparation: CJH AG; manuscript review: all authors. All authors read and approved the final manuscript.

## Supporting information


**Data S1.** Additional information regarding study methods.
