# Supplementary material for: Creating HIV risk profiles for men in South Africa: a latent class approach using cross‐sectional survey data
Source: J Int AIDS Soc. 2020 Jun 26;23(Suppl 2):e25518. doi: 10.1002/jia2.25518 (PMC7319107; doi:10.1002/jia2.25518)
Supplement: Supplementary file 1 — Data S1. Additional information regarding study methods. [file JIA2-23-e25518-s001.docx]

**Supplemental file 1. Additional information regarding study methods**

**Participant recruitment and interview methods**

The target sample size was 800, not including pilot surveys (which, due to their high quality of data, were included in the final sample). Surveys were completed with all eligible men who consented to participate, with a target sample size of 20-30 respondents per site. Hot spot venue procedures generally followed the Priorities for Local AIDS Control Efforts (PLACE) method [1,2]. Hot spot surveys were conducted from Monday-Saturday, at varying times of day based on when the site was busiest. HIV service site surveys were conducted Monday-Friday, from 7:00am-4:00pm. On-site recruitment procedures were as follows. At hot spots, the interviewer approached the nearest potentially eligible participant (i.e., male approximately age 20-40), and, after completing an interview with an eligible and consenting participant, moved to a different area of that venue/site to recruit the next. At HIV service sites, the interviewer approached potentially eligible participants as they arrived in the waiting room, after they had checked in. The purpose of the survey was described to potential participants as learning more about men’s relationships with women, and their use of HIV testing and other services, in order to help improve HIV prevention programs.

Interviewers were approximately half male and half female, African Zulu-speaking, and similar in age-range to participants. To maintain privacy, eligible participants were asked to move to a study tent next to the hot spot venue/HIV service site. Participants were provided non-alcoholic refreshments and were compensated (at approx. $5) if their participation required travel; those recruited at service-based sites were also compensated for staying at the site longer than for their appointment, with a small gift worth about 40 Rand (about $3.20).

**Data analysis approach**

Five profiles were roughly defined as: older married/cohabiting with high income and younger partners (anticipated to be moderate risk); young university student/recent graduate (moderate-risk); industry/informal worker (high-risk); older poor (low-risk); in committed relationship (low-risk). We chose not to construct separate LCA models for risk of HIV acquisition vs. transmission in part because it was not advisable to restrict samples by self-reported HIV status. Information about HIV status, which would be needed to construct separate samples for acquisition vs. transmission models, was self-reported in this study and only 84 men (8.9% of the sample) reported being HIV-positive. This sample size is suboptimal for LCA [3], precluding a separate HIV transmission model. It also likely represents underreporting, since actual HIV prevalence among men ages 20-40 is closer to 23% (national 2017 estimate) [4] or even 35% (KZN 2015 estimate) [5] — likely due to a combination of lack of status awareness and not reporting a known positive status. Either way, restricting samples by self-reported HIV status could bias findings.

**Model identification and fit statistics**

To establish model identification, each model was run with 100 random start values to confirm that the smallest log-likelihood value was modal [6]. For relative-fit statistics, we calculated the Akaike Information Criteria (AIC), Bayesian Information Criteria (BIC), and entropy, and considered the lowest relative AIC and BIC values, and entropy closest to 1.0, to represent better fit [6,7]. Assessment of the conditional independence assumption was conducted as well [6]. For postestimation analyses we reviewed classification accuracy diagnostics (entropy, AvePP, OCC), ensuring they met common cutoff criteria [7,8].

**Sensitivity analyses for sample without HIV-positive men**

We conducted a sensitivity analysis in which we restricted the sample to respondents who did not report being HIV-positive. This analysis resulted in four markedly similar classes to the full-sample model, with item response probabilities differing by only 1-2% (data available upon request). Prevalence of each class increased slightly among the younger classes and decreased slightly among the older, given that more HIV-positive respondents were in the older classes. These results led us to retain the full-sample LCA model.

**References**

1. MEASURE Evaluation. PLACE: Priorities for Local AIDS Control Efforts. Available at: http://www.cpc.unc.edu/measure/tools/hiv-aids/place. 2016.

2. Reynolds Z, Gottert A, Luben E, Mamba B, Shabangu P, Dlamini N, et al. Who are the male partners of adolescent girls and young women in Swaziland? Analysis of survey data from community venues across 19 DREAMS districts. PloS one. 2018;13(9):e0203208.

3. Lubke GH, Luningham J. Fitting latent variable mixture models. Behaviour research and therapy. 2017;98:91-102.

4. Simbayi L, Zuma K, Zungu N, Moyo S, Marinda E, Jooste S, et al. South African National HIV Prevalence, Incidence, Behaviour and Communication Survey, 2017: towards achieving the UNAIDS 90-90-90 targets. 2019.

5. Kharsany AB, Cawood C, Khanyile D, Lewis L, Grobler A, Puren A, et al. Community-based HIV prevalence in KwaZulu-Natal, South Africa: results of a cross-sectional household survey. The Lancet HIV. 2018;5(8):e427-e37.

6. Collins LM, Lanza ST. Latent class and latent transition analysis: With applications in the social, behavioral, and health sciences: John Wiley & Sons; 2010.

7. Celeux G, Soromenho G. An entropy criterion for assessing the number of clusters in a mixture model. Journal of classification. 1996;13(2):195-212.

8. Nagin DS, NAGIN D. Group-based modeling of development: Harvard University Press; 2005.
